# Guided Self-rehabilitation Contract vs conventional therapy in chronic stroke-induced hemiparesis: NEURORESTORE, a multicenter randomized controlled trial

**DOI:** 10.1186/s12883-019-1257-y

**Published:** 2019-03-12

**Authors:** Jean-Michel Gracies, Maud Pradines, Mouna Ghédira, Catherine-Marie Loche, Valentina Mardale, Catherine Hennegrave, Caroline Gault-Colas, Etienne Audureau, Emilie Hutin, Marjolaine Baude, Nicolas Bayle, the Neurorestore Study Group, Jean-Michel Gracies, Alain Yelnik, Patrick Dehail, Philippe Marque, François Boyer, Pascal Giraux, Leila Tlili, Marylène Jousse, Xavier De Boissezon, Gaël Belassian, Paul Gassie, Alice Aguirre, Diego Agueros, Diane Rimaud, Mohamed Tarri

**Affiliations:** 1 EA 7377 BIOTN, Laboratoire Analyse et Restauration du Mouvement, Université Paris Est Créteil (UPEC), F-94010 Créteil, France; 2 AP-HP, Service de Rééducation Neurolocomotrice, Unité de Neurorééducation, Hôpitaux Universitaires Henri Mondor, F-94010 Créteil, France; 3 AP-HP, Service de Santé Publique, Hôpitaux Universitaires Henri Mondor, F-94010 Créteil, France; 4 DHU A-TVB, IRMB- EA 7376 CEpiA (Clinical Epidemiology And Ageing Unit), Université Paris Est-Créteil, F-94010 Créteil, France

**Keywords:** Stroke, Chronic hemiparesis, Self-rehabilitation, Plasticity, Motor function

## Abstract

**Background:**

After discharge from hospital following a stroke, prescriptions of community-based rehabilitation are often downgraded to “maintenance” rehabilitation or discontinued. This classic therapeutic behavior stems from persistent confusion between lesion-induced plasticity, which lasts for the first 6 months essentially, and behavior-induced plasticity, of indefinite duration, through which intense rehabilitation might remain effective. This prospective, randomized, multicenter, single-blind study in subjects with chronic stroke-induced hemiparesis evaluates changes in active function with a Guided Self-rehabilitation Contract vs conventional therapy alone, pursued for a year.

**Methods:**

One hundred and twenty four adult subjects with chronic hemiparesis (> 1 year since first stroke) will be included in six tertiary rehabilitation centers. For each patient, two treatments will be compared over a 1-year period, preceded and followed by an observational 6-month phase of conventional rehabilitation. In the experimental group, the therapist will implement the diary-based and antagonist-targeting Guided Self-rehabilitation Contract method using two monthly home visits. The method involves: i) prescribing a daily antagonist-targeting self-rehabilitation program, ii) teaching the techniques involved in the program, iii) motivating and guiding the patient over time, by requesting a diary of the work achieved to be brought back by the patient at each visit. In the control group, participants will benefit from conventional therapy only, as per their physician’s prescription.

The two co-primary outcome measures are the maximal ambulation speed barefoot over 10 m for the lower limb, and the Modified Frenchay Scale for the upper limb. Secondary outcome measures include total cost of care from the medical insurance point of view, physiological cost index in the 2-min walking test, quality of life (SF 36) and measures of the psychological impact of the two treatment modalities. Participants will be evaluated every 6 months (D1/M6/M12/M18/M24) by a blinded investigator, the experimental period being between M6 and M18. Each patient will be allowed to receive any medications deemed necessary to their attending physician, including botulinum toxin injections.

**Discussion:**

This study will increase the level of knowledge on the effects of Guided Self-rehabilitation Contracts in patients with chronic stroke-induced hemiparesis.

**Trial registration:**

ClinicalTrials.gov: NCT02202954, July 29, 2014.

## Background

The most common motor deficit following stroke is spastic hemiparesis [1]. More than 90% of patients with hemiparesis recover some lower limb function after a stroke, but rarely with a level of ease or speed that would allow for independent and comfortable ambulation in everyday life, outdoors in particular [1–3]. In the upper limb, the proportion of patients that recover daily use of the arm is estimated between 10 and 30% [4–8]. Consequently, around half of stroke survivors do not resume professional activities, and two thirds remain chronically disabled [9].

In parallel, most patients in chronic stages have their rehabilitation discontinued or converted into “maintenance” therapy, as professionals often estimate that they might no longer progress [7, 10–15]. Others benefit from reinduction periods, prescribed according to subjective or ill-defined criteria. It has not been demonstrated that this conventional rehabilitation system now fits current knowledge on behavior-induced brain plasticity and on the potential for motor recovery in chronic spastic paresis [16–18]. Indeed, a significant body of evidence demonstrates that high intensity of rehabilitation (the opposite of “maintenance therapy”) correlates with motor function improvement in chronic stages [16, 19, 20]. One way to achieve sufficient amounts of physical treatment might be to adequately *guide and motivate* the patient into practicing self-rehabilitation [18, 20]. It has been confirmed that programs of exercises given by the therapist to be performed at home are appreciated by patients not only for the structure they give to everyday life, but also as they represent in themselves a source of motivation and hope, particularly when these programs are associated with ongoing professional support [21, 22].

We hypothesize that there is confusion between the lesion-induced plasticity of the central nervous system – essentially during the first 6 months post-lesion – and the behavior-induced plasticity, which lasts indefinitely [16, 17, 23–27]. The latter justifies initiatives to organize chronic and intense physical rehabilitation work [17, 18, 23–28]. Even though previous, short-term open studies evaluating self-rehabilitation programs in spastic hemiparesis suggested the possibility of functional improvement, to our knowledge there are no large-scale prospective randomized controlled protocols that test the effectiveness of long term self-rehabilitation programs in spastic hemiparesis as against conventional rehabilitation systems, especially in chronic stages [29–36].

Technically, which home rehabilitation exercises might be recommended? From a neurophysiological point of view, muscle overactivity chronologically emerges as the third fundamental feature of motor impairment that begins in the subacute phase in hemiparesis, following paresis and soft tissue contracture that appear in the acute phase [37–39]. One recognizable form of muscle overactivity is spasticity (hyper-reflectivity to phasic stretch), which is potentiated by muscle shortening [37, 38]. Hypersensitivity to stretch in an antagonist muscle also impedes voluntary motoneurone recruitment for the agonist muscle, a phenomenon called “stretch-sensitive paresis” [40]. As none of the three fundamental mechanisms of motor impairment (paresis, muscle shortening, and muscle overactivity) is distributed symmetrically between agonists and antagonists, there are force imbalances around joints, hindering active movements and deforming body postures [41]. Each of these three mechanisms of impairment, particularly the two most important, which are muscle shortening and muscle overactivity, can be specifically targeted with local treatment, muscle by muscle, aiming to rebalance forces, joint by joint [28]. For the less overactive muscles around each joint, an intensive motor training will aim to break the vicious cycle Paresis-Disuse-Paresis [37]. For their shortened and more overactive antagonists most importantly, a daily program of self-stretch postures at high load combined with a program of maximal amplitude rapid alternating movements, potentially associated with botulinum toxin injections, will aim to increase muscle extensibility and reduce cocontraction, breaking the vicious cycle: Muscle shortening-Overactivity-Muscle shortening [28, 42, 43] (www.i-gsc.com). Significant preliminary results obtained using prescription and teaching of self-rehabilitation programs within a Guided Self-rehabilitation Contract (GSC) led us to hypothesize that this method practiced over the long term might enhance active motor function in chronic hemiparesis beyond 1 year following stroke [18, 44–48].

From a social point of view, stroke is the leading cause of acquired disability in Western countries. For the Steering Committee on Stroke Prevention and Management in France, the yearly cost of stroke is €5.9 billions, the cost of care in medical and social facilities is €2.4 billions and the cost of daily allowances and disability pensions is €125.8 millions [49]. Additionally, several studies have shown that indirect costs were proportional to direct costs [50]. Stroke thus accounts for a large share of health expenditures. In that regard as well, devising a feasible and effective guided self-rehabilitation program might offer financial advantages for our health systems.

## Objectives

The primary objective of this study is to evaluate changes in lower limb and upper limb motor function after 1 year of Guided Self-rehabilitation Contract compared to conventional therapy alone, at a chronic stage following stroke.

The secondary objectives include the evaluation of: i) quality of life at the end of 1 year with each of the two treatment modalities; ii) direct socioeconomic costs of the two physical treatments from the point of view of the health coverage system; iii) persistence of lower limb and upper limb motor function changes 6 months after the end of the one-year experimental study period; iv) amount of self-rehabilitation in the experimental group over 12 months; v) current, real-life amounts of conventional rehabilitation provided for chronic hemiparesis in the French health system. vi) psychological and occupational therapy endpoints with the two treatments, for a sub-group of 44 patients.

## Methods

### Ethical approval and trial registration

The *Neurorestore* study is carried out in compliance with the Helsinki Declaration. Local ethics committees (CPP Ile-de-France VI, Groupe Hospitalier Pitié Salpêtrière) approved the study protocol (first version, 2010), patient information letter, and informed consent form. Written consent to participate in the protocol was always signed directly by the patient. The *Neurorestore* study is registered in the ClinicalTrials.gov database (NCT02202954, July 29, 2014).

### Research design

**Fig. 1:**
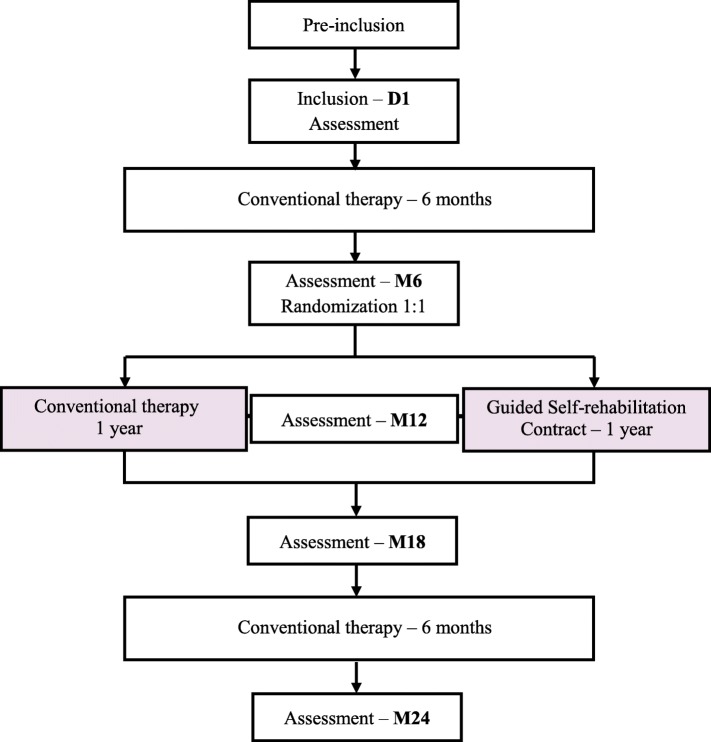
Study protocol

Neurorestore is a prospective, controlled, randomized, multicenter, single-blind study on 124 participants with chronic spastic hemiparesis (> 1 year post stroke) in six French tertiary rehabilitation centers. For all participants, the study will begin with an initial 6-month follow up phase with conventional therapy, to assess its socio-economic costs and the stabilization of the clinical status of stroke patients at a chronic stage in the current rehabilitation system (Fig. 1). The second phase will be the randomized and comparative phase, assessing Guided Self-Rehabilitation Contract (GSC) versus conventional therapy (CONV) alone, over 1 year. It is important to note that in the GSC group, participants will be free of continuing whichever additional treatment they wish, including conventional therapy as prescribed by their physicians. At the end of this comparative period, each participant will be followed for a second, final 6 month follow-up phase with conventional therapy, exactly as in the initial phase. This final phase will explore the persistence of possible differences between the two groups. The total duration of study participation for each subject is 2 years. Data will be analyzed based on intention-to-treat and per-protocol analyses.

### Intervention

#### Conventional therapy

In the Conventional Physical Therapy group (CONV), physiotherapy sessions are freely prescribed by the patient’s attending physician, neurologist or rehabilitation physician, according to medical opinion and patient requests. These community-based therapy sessions are universally and indefinitely covered by the public health insurance in France. Physiotherapists providing rehabilitation sessions in this group will be freely selected by the patient and/or recommended by the physician, as in routine practice.

#### The Guided Self-rehabilitation Contract

**Fig. 2:**
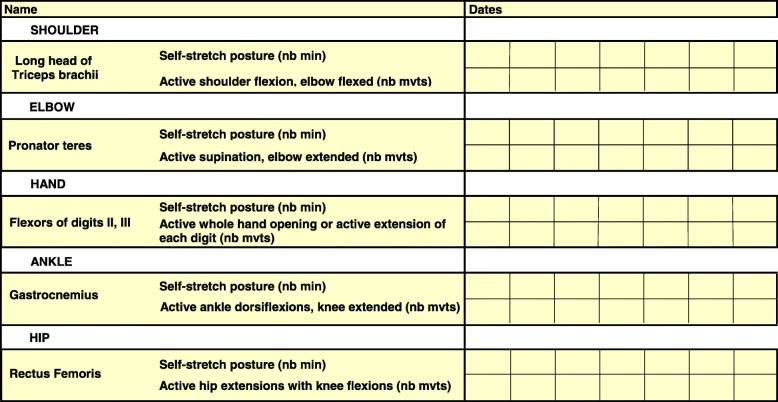
Template of diary in Guided Self-rehabilitation Contract

In the GSC group, patients will be free of following any conventional therapy sessions as in the CONV group. In addition, in each of the six centers, a physiotherapist funded by the study will provide 1.5-h home sessions once every 15 days to:

- Explain the principles of the Guided Self-Rehabilitation Contract to the patient. The GSC is a diary-based and antagonist-targeting rehabilitation system centered on a moral contract in which each party, patient and therapist, commits to each other on the following actions. The therapist commits to:
  - *Prescribe* and *teach* a daily program of self-stretch postures and training exercises appropriate for the patient, and correct the techniques and re-adjust the prescribed program according to patient progress. The therapist will provide a manual or access to a web-based application to the patient, which contains the prescribed stretching and exercise program, with illustrations of the self-stretch postures and training exercises [28], (www.i-gsc.com). In addition, coming to each participant’s home at each visit, the therapist will be able to adapt the various techniques included in the program to the environment of the patient. The stretching program involves static postures of self-stretch at high load (while remaining below the pain threshold) for specific antagonists selected by the therapist. The patient should keep a strong and constant tension on the stretched muscle for a cumulative period of ≥10 min a day per targeted muscle. The training program consists of series of unassisted rapid alternating efforts or movements of maximum amplitude against each targeted antagonist in a short time (e.g. 15 to 30 s per series, depending on fatigability) so as to gradually reduce antagonist co-contraction over time [40, 42, 43].
- *Request a diary* from the patient at each visit, in which the daily time of actual practice of self-stretch postures and the number of efforts or movements carried out at each series of rapid alternating movements in the interval between two visits of the therapist should be noted daily (Fig. 2). The therapist explains to the patient that this *self-monitoring* through the diary actually *belongs* to the therapy, in other words that the same physical exercises without maintaining the diary are likely to not have the same effects [51–60].
  Indeed, written feedback from the patient to the therapist provides substantial benefits such as an increased accuracy of the information returned to the therapist and therefore an easier and more precise coaching task on the therapist’s part, an improved compliance to the self-rehabilitation program and thus enhanced efficacy of this program [51–56]. Finally and most importantly, the diary provides the patient with a positive reinforcement, with potentially even antidepressant effects [57–59]. Regardless of self-monitoring, quantitative feedback on performance provided to the patient has been shown to improve rehabilitation effectiveness during the subacute phase of stroke [60].
- *Verify* patient compliance to the prescribed active training exercises and self-stretch postures by ensuring that the self-rehabilitation diary is well kept. The therapist will count the number of filled out days of the diary and divide it by the number of elapsed days since the last visit. This filling rate is communicated every 15 days to a referring coordinator (one for each study center).

### Outcome measures

To evaluate motor function beyond 1 year after stroke, the two co-primary outcome measures are as follows:

- For the lower limb: capacity of ambulation, assessed by the maximal ambulation speed barefoot without technical aids over 10 m, starting and ending in a seated position (re-assessed by a blinded investigator based on video recordings) [61]. This parameter is correlated with most of the kinematic parameters of gait in hemiparesis and characterized by high intra- and inter-rater reliability [61–64]. Finally, ambulation tests over 10 m (AT10) have strong ecological validity, with respect to the use of walking in natural environments [65–67].
- For the upper limb: the Modified Frenchay Scale (MFS) [68, 69]. This scale is a modification of the Frenchay Arm Test, that consisted in a binary pass-fail rating of seven unimanual activities of daily life [4]. The MFS consists of video-recording ten activities of daily life (4 uni-manual activities using the paretic hand and 6 bimanual activities, in which the paretic hand assists the other hand) and rating them on a ten-point visual analog scale based on the video-review. The maximal score for each task is 10 and the total score is the mean of the ten scores. The rating of each task of the Modified Frenchay Scale demonstrated excellent intra- and inter-reliability and MFS has also been validated against a well-known subjective scale of perceived function (Disability Assessment Scale, DAS) [70, 71]. The study will use a central, a single blind investigator for MFS rating of all visits for all study participants.

The secondary outcome measures are:

1. Ambulation speed, step length and cadence during the ambulation test over 10 m (AT10) barefoot at comfortable speed without technical aid, and step length and cadence during AT10 at maximal speed [61];
2. Ambulation speed, step length, cadence and physiological cost index over a 2-min ambulation test at maximal speed without technical aid [72];
3. Subjective self-assessment of perceived function (DAS) indicating the degree of functional incapacity evaluated by the patient for hygiene and dressing activities, pain and cosmesis [70, 73];
4. Global test of functional dependency (Barthel Index) [74];
5. Quality of life (SF 36) [75, 76];
6. Anxiety and depression test (Geriatric Depression Scale, GDS) [77];
7. Evaluation by questionnaires, completed by the patient (and/or a third person), on the weekly frequency and duration of the conventional rehabilitation sessions and the amount of professional help received at home during the study period, to estimate the direct costs from the point of view of the health coverage system;
8. Estimation of the total cost of healthcare, including medical, socio-medical, allowance cost, combining the points of view of the medical insurance and of the State, to encompass all paying institutions;
9. Evaluation of the amount of self-rehabilitation in the GSC group, through the filling ratio of diaries over 12 months [28], (www.i-gsc.com);
10. Evaluation of functioning and home adaptation by an occupational therapist and by questionnaires, in a sub-group of 44 participants in two of the six study centers;
11. Evaluation of the psychological adjustment of the patient, by a study psychologist in the same sub-group of 44 participants in two of the six study centers, to assess the required psychological resources and the need for psychological support. Each of these participants will be contacted for a first visit and offered to consult with the psychologist for the two-year study period. The frequency of encounters will be adjusted to the need of each patient.

### Setting and recruitment

This multicenter trial involves six French centers (the medical centers in Saint-Etienne, Reims, Bordeaux, Toulouse, and Henri Mondor and Lariboisière university hospitals in Paris). Each of these centers admits > 100 cases/year of patients with moderate to severe stroke-induced hemiparesis for specialized rehabilitation and will recruit 20 to 24 patients. A 3-year time-frame is planned for the study, each center starting the study once the total expected number of participants to be enrolled are lined up. The objective is thus to run the protocol simultaneously for all participants of each center, to make optimal use of the study therapist’s time (GSC group).

### Procedures

In practice, each center uses two investigators in addition to the physical therapist for the GSC group. One is a clinical assessor (physician or physiotherapist) who remains blinded to the participant group; the other is the coordinator whose role is to organize the five semi-annual evaluations at D1, M6, M12, M18, M24. The coordinator will be unblinded and will be in contact with the participant’s physicians and with the physiotherapist involved in the GSC for the study center (for those participants in the GSC group). The coordinating investigator, after receiving the result of the randomization at the M6 visit, will:

- inform the participant and the physiotherapist who will apply the GSC method, for those participants randomized in the GSC group,
- notify the participant to continue with the usual therapy without further details, for those participants randomized in the CONV group.

Pre-inclusions will take place during a regular clinic visit. Participants meeting the selection criteria will be invited to participate in the protocol. A written descriptive documentation will be provided to them and potential study subjects will have at least 2 weeks to decide about participation. The informed consent will be signed and collected on the Day 1 visit of the study.

Phase 1 will then be a 6-month prospective follow-up period - with visits at D1 and M6 (Month 6) - to evaluate any changes under conventional community-based therapy. The same blinded investigator will evaluate participants at all follow-up visits, D1, M6, M12, M18, M24 (Table 1). At each visit, specific attention will be paid to any signs suggesting recurrent strokes with, if necessary, additional prescription of brain imaging. Should there be a diagnosis of recurrent stroke since study enrolment (calling for neuroradiologist opinion if needed), study participation will be discontinued and an Adverse Events form and Study Termination form will be filled out.

**Table 1:** Template of recommended content for the schedule of enrolment, interventions, and assessments

	D1	M6	M12	M18	M24
Verification of inclusion and non-inclusion criteria	x				
Signature of written consent	x				
Full neurologic assessment	x				
Randomization		x			
Inventory of concomitant, local and systemic medical treatment s	x	x	x	x	x
Inventory of technical aids	x	x	x	x	x
Inventory of patient suppor t (caregivers, equipment)	x	x	x	x	x
Comfortable and maximal ambulation speed over 10 m s	x	x	x	x	x
Endurance 2-min walking test – Physiological Cost Index	x	x	x	x	x
Modified Frenchay Scale	x	x	x	x	x
Disability Assessment Scale (DAS)	x	x	x	x	x
Barthel Index	x	x	x	x	x
Quality of Life scale (SF36)	x	x	x	x	x
Geriatric Depression Scale	x	x	x	x	x
Questionnaires to patient/caregiver on frequen cy of therapy sessions	x	x	x	x	x
Occupational therapy assessment (in subgroup of 44 patients)		x		x	
Clinical psychologist assessment (in subgroup of 44 patients)	x	x		x	

Throughout the study, participants will be permitted to freely use any “antispastic” medication such as systemic synaptic depressor drugs (baclofen, benzodiazepines, dantrolene sodium, tizanidine) or botulinum toxin injections as well as any technical aid deemed necessary to their attending physician or physiotherapist. However, it is recommended to taper systemic antispastic agents as much as possible (or taper them off) during the first 6-month follow-up phase because of their well-documented antiplasticity and anti-recovery effects [78–81]. If participants are still under systemic depressors at the onset of the randomized study phase, doses should then be kept constant as much as possible.

After the first 6 months of follow-up, participants will be randomized into two groups: “conventional therapy” (CONV) and “Guided Self-rehabilitation Contract” (GSC) (see Randomization).

In order to minimize any nocebo effect in the conventional treatment group, the referent coordinator will not disclose the details of the Guided Self-rehabilitation Contract to the physicians and physiotherapists who treat participants in the CONV group, nor to the CONV participants themselves.

### Randomization procedure

The randomization list will be computer-generated by a statistician from the Clinical Research Unit of Paris-Est Créteil University, independent from the study. The randomization number for each participant will be requested by the evaluating investigator (blinded) at the end of Visit 2 (M6) and then transmitted electronically to the referring coordinator (unblinded) of the center. Each participant will be randomized into one of the two treatment groups: the CONV group (Conventional Therapy) and the GSC group (Guided Self-rehabilitation Contract). Randomization will be stratified by center.

### Study population

To participate in this study, subjects must have the decision making capacity to give informed written consent based on the investigator’s judgment, and meet all of the inclusion criteria and none of the exclusion criteria listed below.

#### Inclusion criteria

1. Hemiparesis due to stroke, for over a year before enrolment;
2. Age > 18 years;
3. Ability to ambulate over 10 m independently, barefoot and without technical aid;
4. Maximal 10-m ambulation speed between 0.1 and 1.3 m/sec;
5. Modified Frenchay Scale score > 2/10 and < 8/10;
6. Written consent to participate in the protocol, signed by the patient

#### Exclusion criteria

1. Recurrent stroke;
2. Significant orthopedic disorder in lower limb;
3. Cognitive, phasic or behavioral dysfunction affecting patient participation;
4. Non affiliation to medical insurance system.

### Statistical methods

#### Sample size

With respect to the Modified Frenchay Scale, based on an average score of 4.4 ± 1.9 in a similar population, [82] the alternative hypothesis (H1) is an improvement of 30% in the GSC group versus a 10% improvement in the CONV group. To demonstrate this hypothesis with a bilateral 80% power and an alpha score of 5%, *n* = 62 subjects per group are required for a total of 124 participants.

With respect to the maximal ambulation speed over 10 m, with an assumed baseline ambulation speed of 0.63 ± 0.43 m/sec and with the hypothesis of a 50% improvement in the GSC group versus a 10% improvement in the CONV group, at least *n* = 47 participants per group are required using a bilateral power of 80% with an alpha of 5% [47]. A total of 124 patients will thus be included in the protocol. This number should also allow sufficient power to evaluate secondary criteria, including a description of the monthly frequencies of the rehabilitation sessions, of the required aids and allowances and therefore an estimation of the cost from the point of view of the health insurance and the State.

#### Statistical analysis

This study will require several statistical analyzes. The main analysis will focus on the randomized phase of the study (from M6 to M18).

#### Descriptive analysis of the first phase

For Phase 1 (Day 1 to M6), descriptive analysis will explore:

- participant characteristics: age, sex, etiology of stroke, presence of significant sensory deficit, apraxia, neglect at Day 1 and M6.
- changes in function over the first 6 months of follow-up: ambulation speed, upper limb function (MFS) and secondary criteria.
- the current conventional therapy treatment: frequency of sessions with the community-based physiotherapist, number of visits and costs. The sum of the monthly frequency during the first 6 months of rehabilitation and the monthly cost of these sessions will be estimated for all 124 participants together. Comparisons will then be made between different levels of severity at onset and different age groups.

#### Analysis of the randomized study

The analysis of the randomized study will be carried out based on intent to treat and per protocol.

The primary and secondary criteria and their changes from M6 to M18 will be compared between the two groups using mixed models. The changes will also be compared at M24, i.e. 6 months following the end of the study treatment. These same analyzes will be carried out by subgroups, comparing the availability of a psychological follow-up or not, ie in the participants recruited by the Mondor-Lariboisière centers versus those of the other centers.

A multivariable analysis will be carried out by considering the characteristics of the patients (age, sex), the center, the initial severity parameters of the disease (sensory disturbances) or time since stroke, as well as the availability or not of a psychologist.

#### Pre−/post-analysis

With patients being their own control, the changes in various functional parameters during the first 6 months of follow-up with conventional therapy (from Day 1 to M6) will be compared with the changes in these same parameters:

- during the randomized study between M6 and M12
- during the randomized study between M12 and M18
- and during follow-up between M18 and M24.

The mean changes and the rate of progression over periods of 6 months will be compared. These same comparisons will be realized in subgroups according to the randomization group, but also depending on any specific psychotherapeutic management (for 44 participants, in the two centers involved).

#### Statistical tests and software used

For comparisons between selected subgroups on the basis of clinical starting variables or demographic data, statistical analyzes will use:

- for continuous variables: comparison of means by Student test (*t*-test) or *t*-test for paired data, and in case of absence of normality, using nonparametric tests: Wilcoxon test for comparison of 2 measurements (Wilcoxon test for independent series or Wilcoxon test for matched series according to the analysis, or Kruskall-Wallis test for the comparisons of more than 2 measurements).
- for qualitative variables: X^2^ of Pearson or that of Mantel-Haenszel; X^2^ of Mac Nemar for matched series, according to the analyzes carried out.

The analyzes will be carried out by the Clinical Research Unit of Hôpital Henri Mondor, using the R 2.13.0 software.

### Safety

The Ethics Committee will notify the sponsor (DRCD-APHP) before research is started. Furthermore, to comply with the French regulations, the declaration of digital files of personal data to be collected for the study will be completed before the actual study onset. Therefore, implementation of data collection and processing will be subject to prior approval of the Advisory Committee on Research Information in the Field of Health (‘CCTIRS’) and the National Commission for Information Technology and Freedom (‘CNIL’). Finally, “Assistance Publique des Hôpitaux de Paris” (APHP), sponsor of this research, has contracted an insurance for the duration of the research, in compliance with the law on biomedical research, guaranteeing its own civil liability as well as that of any intervening physician or staff involved in carrying out the research.

## Discussion

A number of technical and psychological differences may be anticipated between the two treatments. Technically, in the rehabilitation techniques used in current practice, emphasis is usually not on the practice of rapid alternating movements, particularly of maximal amplitude, while the effectiveness of this method and its physiological mechanisms (restoration of reciprocal inhibition allowing for the gradual reduction of cocontraction) have been established [42–44]. Furthermore, in recent literature, pessimistic conclusions have been common regarding the effectiveness of stretch, while no long term (> 6 months) controlled study on stretch is yet available in the literature in spastic paresis [18, 83]. In any case, conventional therapy usually does not involve the daily duration of passive muscle stretching recommended in the Guided Self-rehabilitation Contract method (at least a cumulative 10 min of submaximal stretch postures per muscle per day), and as a consequence, this amount of stretch might not be sufficient to allow muscle plasticity [18, 84]. On a psychological note, patient responsibilisation is the essence of the Guided Self-rehabilitation Contract system. The patient has an active responsibility to accomplish the prescribed daily work and to notify it in the diary, in stark contrast with the common passive expectation of “recovery” with community-based therapy sessions in current practice [28, 85, 86].

Overall, this study will largely increase the level of knowledge on the effects of Guided Self-rehabilitation Contracts and on the rehabilitation access for patients with chronic stroke-induced hemiparesis patients. Should Guided Self-rehabilitation Contracts prove more beneficial than the conventional community-based rehabilitation system, the duration and frequency of the rehabilitation sessions provided by the therapist could be adjusted at a chronic stage, decreasing the direct cost of cares of our insurance system [49, 50, 86, 87].

## Abbreviations

APHP: Assistance Publique des Hôpitaux de Paris
AT10: Ambulation Test over Ten meters
CCTIRS: Comité Consultatif sur le Traitement de l’Information en Matière de Recherche dans le Domaine de la Santé
CNIL: Commission Nationale de l’Informatique et des Libertés
CPT: Conventional Physical Therapy
DAS: Disability Assessment Scale
DRCD: Département de la Recherche Clinique et du Développement
GSC: Guided Self-rehabilitation Contract
MFS: Modified Frenchay Scale
